# Evaluation of a Simplified Method for GC/MS Qualitative Analysis of Polycyclic Aromatic Hydrocarbons, Polychlorinated Biphenyls, and Organic Pesticides Using PARADISe Computer Program

**DOI:** 10.3390/molecules25163727

**Published:** 2020-08-15

**Authors:** Łukasz Dąbrowski

**Affiliations:** Department of Food Analysis and Environmental Protection, Faculty of Chemical Technology and Engineering, UTP University of Science and Technology, 3 Seminaryjna Street, 85-326 Bydgoszcz, Poland; lukas@utp.edu.pl; Tel.: +48-523-749-014

**Keywords:** PARADISe software, data processing, mass spectra deconvolution, GC/MS

## Abstract

For complex matrices such as environmental samples, there is usually a problem with not fully resolved peaks during GC/MS analysis. The PARADISe computer program (based on the PARFAC2 model) allows the identification of peaks using the deconvoluted mass spectra and the NIST MS library. The number of repetitions required by this software (at least five) is a real limitation for the determination of semi-volatile compounds, like polycyclic aromatic hydrocarbons, polychlorinated biphenyls, and organic pesticides in environmental samples. In this work, the method to overcome this condition was proposed and evaluated. The sets of the five files required by PARADISe were prepared by mathematically modifying the original GC/MS chromatograms obtained for the standard mixture (C = 2 µg/mL of 40 compounds) and real sample extracts (soil samples with different total organic carbon content and one cardboard extract) spiked with standards. Total average match factor for all the substances identified in a standard mixture was 874 (near 900—“excellent match”), and for all the substances in the real samples, it was 786 (near 800—“good match”). The results from PARADISe were comparable to those obtained with other programs: AMDIS (NIST) and MassHunter (Agilent), tested also in this work. PARADISe software can be effectively used for chromatogram deconvolution and substance identification.

## 1. Introduction

Environmental samples are usually a mixture of many different substances. The main problem of substance identification is the coelution of several not fully separated compounds. In this case, there are several options for improving the selectivity of the final analysis method. One of them is the confirmation of GC/MS data by parallel analysis of the sample with a selective detector [1,2], which provides complementary information on the structure of the compound.

In the case of lack of these additional data, qualitative analysis is usually based on GC/MS results only. It is performed using various deconvolution algorithms. Comparative studies of deconvolution computer programs and algorithms have already been described in many papers [3,4,5,6,7]. Among the various programs, AMDIS (automated mass spectral deconvolution and identification system) from NIST serves as a reference for its popularity and free access. Reliability of the results generating by this program (i.e., the effectiveness of identification) depends on the number of operational settings (e.g., deconvolution settings) of the software and GC [3,8,9]. It is known from various experiments that AMDIS tends to produce many false-positive components [3,10]. One of the AMDIS-based software is—commercially available from Agilent—MassHunter (MH) [11]. It allows to find compounds by chromatogram deconvolution, and then identify them by searching MS libraries. It is relatively easy, fast and accurate—without the user having to experiment with the operating settings [12].

Another approach to GC/MS data deconvolution is based on a model called PARAFAC2 (PARAllel FACtor analysis 2) [13,14,15,16]. This model allows the mass spectrum to be extracted for each component by combining information from multiple chromatograms obtained for the same sample. The background can be modelled and separated as one or more components. PARAFAC2 usage is limited to the mathematical users and needs extensive coding [11] (usually in MATLAB). Nevertheless, it has been used in many applications due to its high efficiency of chromatogram deconvolution. It was used, inter alia, to identify and quantify compounds in wine and tobacco [17], volatile organic compound emissions from poultry farm [18], compounds which permeate through the membrane from industrial dairy ingredient production used as process water [19] and others [6,20].

The graphical user interface for PARAFAC2 was developed at the University of Copenhagen and is called PARADISe (PARAFAC2 based Deconvolution and Identification System) [5,11]. Johnsen et al. (2017) characterized the PARADISe system’s limitations and advantages [11]. This computer program is becoming more and more popular recently. It was used for the non-target analysis of volatile compounds in olive oil [21], wood smoke volatile composition [22], products from pyrolysis of poplars [23], and composition of cashew apple juice [24].

One of the requirements of the PARADISe system is that “at least five samples with independent variations must be included in the sample set” [11]. It is a real limitation in semi-volatile environmental pollutants determination (like polycyclic aromatic hydrocarbons: PAHs, polychlorinated biphenyls: PCBs, organic pesticides) to obtain more than several replicates of the sample. Soil, sediment, river water, and other samples contain—even after a thorough cleaning—many different substances that can contaminate the GC/MS system. This leads to progressive deterioration in instrument performance caused by the ion source and optics contamination [12]. Therefore, identification of non-targeted compounds involves a rough analysis of the sample extract, usually based on one to three samples.

In order to be able to use PARADISe in such a case, a simplified method of analyzing the GC/MS data has been proposed, based on a limited set of data derived from one chromatogram. Additional chromatograms were generated by mathematical modifications (shifting the peak retention time and multiplying the signal to simulate “independent variations” of the samples) of the original one to meet the requirements of the software. The aim of this work was to evaluate the usage of PARADISe for the analysis of polycyclic aromatic hydrocarbons, polychlorinated biphenyls, and organic pesticides. The results achieved in this way were compared to the results obtained from AMDIS (NIST) and MassHunter (MH) by Agilent.

## 2. Results and Discussion

**Figure 1:**
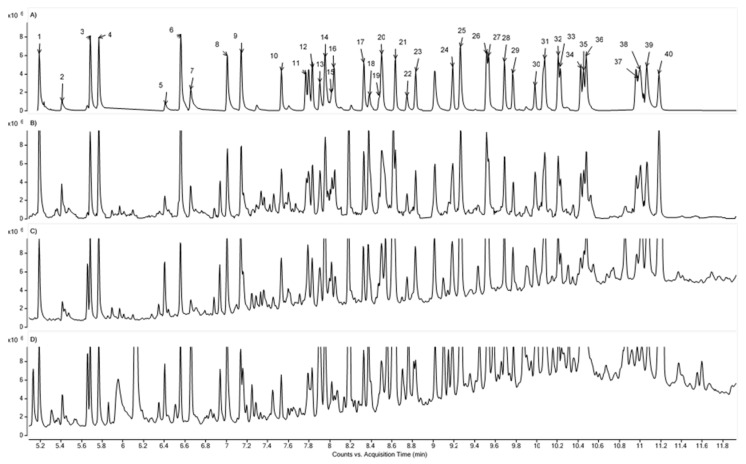
Sample chromatograms of (**A**) the standard mixture (2 µg/mL) and extracts from the samples: (**B**) S1, (**C**) S2, (**D**) S3 with added standards at 2 µg/mL. The labels above the peaks on the chromatogram (**A**) correspond to the No column in Table 1.

Sample chromatograms obtained from the GC/MS analysis of the standard mixture and real samples are shown in Figure 1.

### 2.1. Standard Mixture Analysis

The performance of the PARADISe software was evaluated using standard analyte solutions at the concentration of 2 µg/mL. Mixtures of lower concentrations (1.3 µg/mL, 0.75 µg/mL, and lower), were tested, but many analytes were not found (mainly PAHs and organophosphorus pesticides). A similar trend was also observed when using other deconvolution packages (AMDIS, MassHunter). Further experiments were carried out at the concentration of the standard mixture of 2 µg/mL.

The results obtained from the PARADISe program were presented in Table 1 (the number of repetitions = 3, which is typical for this type of experiments [3,4]). All analytes were correctly identified using PARADISe program.

Due to the similar structure, it was not possible to distinguish some compounds (in any of the tested programs), i.e., isomers of HCHs, PCB congeners with the same number of chlorines, 1-methylnaphthalene and 2-methylnaphthalene or PAHs with the same formula. In this study findings of the compound with the same formula were treated as a positive hit.

Average mass factors (AMFs) and average reversed match factors (ARMFs) were calculated by taking the match factors from all three repetitions of the analysis (Table 1). For fenchlorphos the average match factor is below 700—probably because of the high similarity of its mass spectrum to co-eluting heptachlor (which MS spectrum is more complicated and consists of different ions, partially the same as fenchlorphos). There was also a problem in chrysene detection—only one positive hit. For all other compounds, PARADISe correctly detected them in three replicate analysis with a high match factor, even if the substances were not fully separated (e.g., 4,4′-DDT, endosulfan sulfate, and PCB 137) and their resolution factor was close to zero. According to the NIST MS Library User’s Guide, match factor (MF) can be classified in the following way: “900 or greater is an excellent match; 800–900, a good match; 700–800, a fair match and less than 600 is a very poor match” [25]. The results should also be in agreement for two or more independent analyses [4]. The most numerous group of the results (85%) contains those with AMF above 800. There is no case where AMF is less than 600. Similar criteria, as described above, were applied to the results obtained with the AMDIS software.

For MH, another algorithm for calculating the quality of the spectral match is used, called Score. The result was considered positive if the Score was higher than 70%. By analyzing the results obtained with MassHunter, two compounds were detected with low probability, i.e., anthracene (1 hit) and methoxychlor (MH Score for this compound below 70, Table 1). Problems with the correct identification of these two compounds also occurred when using AMDIS: 2 positive hits (MF > 700).

### 2.2. Real Sample Analysis

Sample chromatograms of the extracts from the samples S1, S2, S3 (with added standards at 2 µg/mL) were presented in Figure 1B–D, respectively. Additional peaks from the sample components and the increase of the baseline level can be observed on chromatograms. Compared to the standard chromatogram of the mixture, an increase in the height of the analyte peaks was also noted. This may be due to coelution with substances present in the extracts, as well as matrix-induced signal enhancement phenomena [26]. For comparison, all chromatograms are on the same OY scale.

The results obtained from PARADISe program are given in Table 1. Average match factors (AMFs and ARMFs) were calculated from the MFs obtained for all replicates of three different real samples. Additionally, the number of positive identifications in three replicate analysis were presented (similarly to the results for standard mixture). Identification of the analytes in more complex mixture leads to reduce detection efficiency in the case of higher organic carbon content (samples S2, S3). Coelution of analytes with large amounts of phthalates, siloxanes (e.g., from pipette tips, column bleed, and other sources) and other substances present in the sample can be observed in real samples. In such cases, the analytes were not identified, due to the overloaded peaks, which should be considered as one component in the PARAFAC2 model [27]. As an example: PCB 180 has very similar retention time (11.18 min) to bis(2-ethylhexyl) phthalate (identified with MF = 805), which creates an overloaded peak. As a result, PCB 180 is often not detected. After diluting the extract fivefold, re-analyzing the sample with GC/MS and increasing the number of components in the model to eight, PCB 180 was identified, but with an MF of only 550 (RMF = 720). In this case, this result should be confirmed by another method of identification.

In the case of two analytes: anthracene and endrin, the AMF is 642. This is below 700 and can be classified as almost as a “fair match” [25]. Average reversed MF for anthracene is quite high and equals 829. There is a problem with the detection of two other compounds: parathion-methyl, chlorpyriphos (AMF: 488 and 474, respectively). This is due to the fact that huge amounts of substances originated from the real samples can be observed on chromatograms at retention times similar to the analytes (Figure 1B–D). Chlorpyriphos was also not detected either with MH (only 1 hit) or AMDIS (no hits). Analyzing the results in Table 1 it can be concluded that PARADISe software provides complementary information to MH or AMDIS. There are cases where PARADISe detects compounds in a more effective way than MH (β-HCH, PCB 180, methoxychlor, anthracene) and AMDIS (β-HCH).

Total average MF for all the substances in a standard mixture is 874 (near 900—“excellent match”), and for all the substances in the real samples, it is 786 (near 800—“good match”) [25]. The calculated difference between the Total ARMFs and AMFs is 43 for standard mixture and 62 for the real samples. With the exception of a few compounds (e.g., acenaphtylene, pyrimethanil, anthracene, d-BHC, parathion-methyl, fenchlorphos, *p,p’*-DDD, where the differences are higher), it can be concluded that these differences (due to background or co-eluting substance) are small and the deconvolution procedure was effective enough.

## 3. Materials and Methods

### 3.1. Materials and Reagents

Dichloromethane (DCM) and acetonitrile (ACN) were of pesticide residue grade from Merck (Poznań, Poland). Aluminium oxide 90 active neutral for column chromatography obtained from Merck (Poznań, Poland) was used as an SPE bed (200 mg).

### 3.2. Preparation of Analytical Standards Solution

Stock solutions of mixtures of pesticides, PCBs and PAHs, were obtained from Supelco (Poznań, Poland). Standard solutions were diluted from the stock solutions with dichloromethane. The concentration of each component in the standard solution was between 0.2 and 2 µg/mL.

### 3.3. Real Sample Preparation

Two types of soil samples were collected. The total organic carbon (TOC) content was different, i.e., in the sample S1: 0.97% and in the sample S2: 4.41%. The soil was dried at 40 °C and sieved (mesh size 0.43 mm) at room temperature. Additionally, a sample of a high concentration of organic matter: cardboard (S3) used as a packaging material for oranges was also taken (cardboards are usually treated with various protective agents and contain post-production organic compounds).

The samples were prepared according to the procedure described earlier, with some modifications [28,29]. Soil samples (3 g) were extracted with DCM (2 × 8 mL) in an ultrasonic bath for 20 min (2 × 10 min). The extract was evaporated under a gentle stream of nitrogen till dry. The dried residue was dissolved in 2 × 300 µL of ACN and cleaned up on alumina (200 mg) SPE bed. A fraction of 1 mL was eluted with acetonitrile, evaporated and dissolved in 300 µL of standard solution (2 µg/mL). The samples were then analyzed using GC/MS.

### 3.4. GC/MS Analysis

The GC–MS analysis was performed using a 7890B gas chromatograph equipped with a 7693 autosampler and a 5977B mass-selective detector (Agilent, Santa Clara, CA, USA). The capillary column used was HP-5MS, 30 m × 0.25 mm × 0.25 mm, from Agilent (Santa Clara, CA, USA). Helium was used as carrier gas at 1.5 mL/min. The split–splitless injector was operated in pulsed pressure splitless mode as follows: initial pressure 0.2 MPa (30 p.s.i.) for 1.3 min, decreased to constant flow. The purge valve was opened after 1.5 min. The injection volume was 5 µL. The temperatures of the GC system were the following: injector temperature 290 °C; transfer line temperature 280 °C; oven temperature program: 50 °C (1.5 min)–30 °C/min–180 °C–20 °C/min–280 °C (20 min). MS detector (quadrupole) was operated in the EI mode at 70 eV with a mass scan range of 50–450 *m*/*z* and the sampling rate of 3.6 scans/s.

### 3.5. Data Processing

Several virtual machines with 12–20 processors (Intel Xeon E312xx or Intel Core i7 9xx, Santa Clara, CA, USA) and 16–32 GB RAM were used for Windows-based applications. All the software was operated with 64-bit Windows 8.1 Enterprise operating system.

#### 3.5.1. PARADISe Program

PARADISe v 3.87 was used for the analysis standard solution, and real sample chromatograms obtained from GC/MS analysis. As mentioned earlier, it is rather uncommon—for environmental samples such as water or soil and sediments—to repeat analysis more than 2–3 times. This would make this software unusable due to few processed chromatograms. When analyzing the variability of the peaks (for one analyte) on GC chromatograms originated from one sample type (several repetitions), it can be observed that there are some differences among them in retention time and, in general, in peak shape (also caused by various concentrations). Such a variation is a necessary condition when using PARADISe software [11]. To artificially achieve such changes, several chromatograms were generated from a single GC/MS chromatogram after some mathematical modifications. The original file (acquired from GC/MS: standard mixtures and spiked extracts from the real samples) was modified with OpenChrom software [30] using multiplier filter and retention time shifter. Multiplier filter with the factors between 0.5 to 1.5 was used. The retention time shifter was applied with offset backwards or forward between 0 and 0.003 min. These values were obtained by observing the retention time deviation of the analytes during the analysis of real samples. Several (usually four) additional chromatograms were created and exported to CDF files. This resulted in the minimum number of chromatograms (five) that were necessary for PARADISe to run. In the case of the real samples, an additional denoising filter with default settings was applied: this made it easier to establish an appropriate number of intervals (Figure S1). Subsequently, all chromatograms (one primary and four derived from it) were opened in PARADISe v 3.87 program and number of the intervals were established manually: for the standard mixture solution—it was about 60 and for the real samples from 93 to 107 (set individually for the sample of the same kind). The number of the intervals was higher than the number of compounds in the standard mixture, because for not fully separated substances, the peaks originating from them were selected in more than one interval: covering several compounds as well as only one, if possible. In this case, during the final analysis of the results, the same substance was discovered twice or more times (with the same retention time). This procedure was used due to the limitation of the PARADISe program: it cannot deconvolute peaks when the data interval contains substances with identical mass spectra—this is the case with not fully separated PAHs.

**Figure 2:**
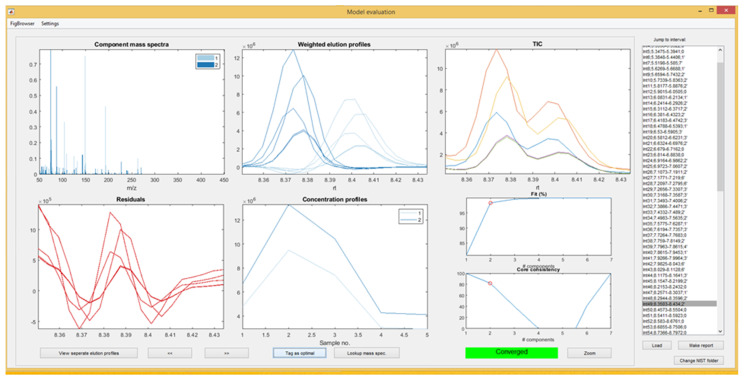
PARADISe model evaluation window with PCB 28 and parathion-methyl peaks (sample S3). Original chromatogram and four additional chromatograms derived from it. The original file was modified with the following parameters, which were generated randomly (retention time shifter [min], multiplier): (−0.00156, 1.99866); (0.00221, 1.56173); (0.00158, 0.63779); (−0.00107, 1.00608).

PARAFAC2 modelling was run with the following parameters: default number of components per interval: from 1 to 7; the maximum number of iterations: 50,000 and with *Non-negativity* field marked. On a twenty-processors (CPUs) virtual machine, the computation was completed in 2–3 h. Models were then evaluated according to the information provided in recently published articles [11,21] and on the YouTube channel QualityAndTechnology [27], which provides step-by-step instructions on how to use this program and interpret the results obtained. Models for each interval were inspected individually. The number of components was established by analyzing all the information provided in the *Model evaluation* window to ensure adequate model fit (over 90%), noise removal, and low residuals, with a core consistency over 90% (Figure 2). With the selected model for each interval, a report was generated (in xls file format) using NIST17 MS library.

#### 3.5.2. AMDIS Program.

For comparison, data analysis was also performed using the AMDIS program (version 2.73, NIST, Gaithersburg, MD, USA) together with the NIST17 MS library. For the AMDIS, the analysis type was used: *Use Retention Index Data* with target library NISTEPA.MSL. The following AMDIS deconvolution settings were used: *Adjacent peak subtraction*: One; *Resolution*: Medium; *Sensitivity*: Very Low; *Shape requirements*: Medium (similar to [3]). The Kovats indices were determined using the mixture of alkanes (C12–C27). AMDIS generated spectra for identified and unidentified compounds. A search of the NIST Library (NIST17) was then performed with a *Minimum match factor* of 75 for all components.

#### 3.5.3. MassHunter Program

In addition, the GC/MS data were also evaluated with MassHunter (MH) Workstation Software Qualitative Analysis Workflows (version B.08.00, Agilent Technologies Inc., Santa Clara, CA, USA). It was operated with the following parameters: compounds were discovered by chromatogram deconvolution with the default settings; substances were identified using MS library (NIST17) search.

## 4. Conclusions

The proposed, simplified method, based on one chromatogram and limited data set generated from it can be efficiently used for the chromatogram deconvolution with the PARADISe software. The results obtained in the proposed procedure are comparable to those form AMDIS or MassHunter. PARADISe can be treated as complementary software to these two programs. Using the software tandem, like PARADISe and MH or PARADISe and AMDIS allows efficient qualitative analysis of the semivolatile pollutants (like PAHs, PCBs, organic pesticides) in the real samples. A high concentration of the matrix components (a common situation in the analysis of the environmental samples) leads to problems with the identification of the compound. In this case, it is necessary to clean up the sample enough to obtain a lower background (various methods, depending on the properties of the analytes, can be helpful for this purpose, mainly different types of solid phase extraction, adsorption chromatography, gel permeation chromatography, sulfuric acid cleanup, and other [28,31,32,33]), then the deconvolution can be an efficient tool in non-target analysis.

## Figures and Tables

**Table 1:** Average NIST MS search match factor (AMF), average NIST MS search reversed match factor (ARMF), and the number of replicates for the standard mixture (std), sample S1 (S1), sample S2 (S2), and sample S3 (S3) for PARADISe computer program, MassHunter (MH), and AMDIS software.

			Standard Mixture					Real Samples										
		RT	PARADISe			MH	AMDIS	PARADISe					MH			AMDIS		
No	Compound	[min]	AMF	ARMF	std	std	std	AMF	ARMF	S1	S2	S3	S1	S2	S3	S1	S2	S3
1	Naphthalene	5.19	945	956	3	3	3	895	928	3	3	3	3	3	3	3	3	3
2	Dichlorvos	5.41	835	884	3	3	3	796	826	2	3	3	3	3	3	3	3	3
3	2-methylnaphthalene	5.68	867	900	3	3	3	893	928	3	3	3	3	3	3	3	3	3
4	1-methylnaphthalene	5.77	922	931	3	3	3	903	924	3	3	2	3	3	3	3	3	3
5	Acenaphthylene	6.41	769	959	3	3	2	697	900	3	3	3	2	2	2	2	2	2
6	Acenaphthene	6.56	942	960	3	3	3	919	935	3	3	3	3	3	3	2	3	3
7	o-hydroxybiphenyl	6.70	888	914	3	3	3	888	905	3	3	3	3	3	3	3	3	3
8	Fluorene	7.01	890	929	3	3	3	888	907	2	3	3	3	3	3	3	3	3
9	PCB 10	7.14	822	887	3	3	3	844	904	3	3	3	3	3	3	3	3	3
10	a-BHC	7.53	948	965	3	3	3	872	907	3	3	3	3	3	3	3	3	3
11	b-BHC	7.76	923	950	3	3	3	698	776	3	3	0	2	1	0	3	3	1
12	Lindane	7.83	920	938	3	3	3	848	889	3	3	2	3	3	3	3	3	3
13	Pyrimethanil	7.92	793	954	2	3	3	701	810	2	3	3	3	3	1	3	3	3
14	Phenanthrene	7.96	928	964	3	3	3	890	930	3	3	3	3	3	3	3	3	3
15	Anthracene	8.01	861	959	3	1	2	642	829	0	2	2	1	3	3	3	3	3
16	d-BHC	8.04	830	920	3	3	3	819	894	3	3	3	3	3	3	3	3	3
17	PCB 28	8.33	910	952	3	3	3	892	904	3	3	3	3	3	3	3	3	3
18	Parathion-methyl	8.39	761	842	3	3	3	488	736	1	0	0	2	1	3	3	3	3
19	Heptachlor	8.50	724	728	3	3	3	683	809	3	1	3	3	3	3	3	3	3
20	Fenchlorphos	8.51	664	728	2	3	3	629	703	3	3	2	3	3	3	3	3	3
21	PCB 52	8.63	944	962	3	3	3	857	877	3	3	0	3	3	0	3	3	2
22	Chlorpyrifos	8.80	900	906	3	3	2	474	467	3	3	0	1	0	0	0	0	0
23	Aldrin	8.83	919	921	3	3	3	745	739	3	3	3	3	3	3	3	3	3
24	Heptachlor epoxide	9.19	887	890	3	3	3	741	792	3	3	3	3	3	3	3	3	3
25	Fluoranthene	9.26	875	938	3	3	3	871	924	3	3	3	3	3	3	3	3	3
26	Pyrene	9.52	857	925	3	3	3	825	911	3	3	3	3	3	3	3	3	3
27	a-endosulfan	9.53	926	929	3	3	3	775	831	3	0	1	3	3	3	3	3	3
28	4,4’-DDE	9.69	948	975	3	3	3	858	914	3	3	3	3	3	3	3	3	3
29	Dieldrin	9.77	930	931	3	3	3	810	830	3	2	3	3	3	3	3	3	3
30	Endrin	9.98	932	940	3	3	3	642	661	3	0	2	3	2	3	3	3	3
31	p,p-DDD	10.10	845	937	3	3	3	829	909	3	3	3	3	3	3	3	3	3
32	PCB 153	10.21	912	954	3	3	3	881	915	3	3	3	3	3	3	3	3	3
33	Endrin aldehyde	10.23	924	926	3	3	3	760	783	2	2	2	2	3	3	3	3	3
34	4,4-DDT	10.43	901	941	3	3	3	727	818	3	2	1	3	3	3	3	3	3
35	Endosulfan sulfate	10.45	864	869	3	3	3	749	772	2	1	0	3	3	3	3	3	3
36	PCB 137	10.48	853	902	3	3	3	801	910	3	3	3	3	3	3	3	3	3
37	Methoxychlor	10.99	732	805	3	3	2	673	818	3	3	3	1	2	3	3	3	3
38	Benz[a]anthracene	11.05	832	898	3	3	3	894	933	3	3	3	2	3	3	3	3	3
39	Chrysene	11.10	881	921	1	3	3	903	933	3	3	3	3	3	3	3	3	2
40	PCB 180	11.18	944	962	2	3	3	747	854	3	1	1	0	2	2	2	3	3

## Supplementary Materials

The following are available online at https://www.mdpi.com/1420-3049/25/16/3727/s1, Figure S1: Effect of denoising filter on chromatograms: five chromatograms (sample S3) imported as CDF files into PARADISe program A) without the denoising filter B) with the denoising filter applied earlier.
