# Supplementary material for: Evaluation of a Simplified Method for GC/MS Qualitative Analysis of Polycyclic Aromatic Hydrocarbons, Polychlorinated Biphenyls, and Organic Pesticides Using PARADISe Computer Program
Source: Molecules. 2020 Aug 15;25(16):3727. doi: 10.3390/molecules25163727 (PMC7465948; doi:10.3390/molecules25163727)
Supplement: Supplementary file 1 [file molecules-25-03727-s001.pdf]

Supplementary materials

Evaluation of a simplified method for GC/MS qualitative analysis of Polycyclic Aromatic Hydrocarbons, Polychlorinated Biphenyls and organic pesticides using PARADISe computer program

Łukasz Dąbrowski

UTP University of Science and Technology, Faculty of Chemical Technology and Engineering, Department of Food Analysis and Environmental Protection, 3 Seminaryjna Street, 85-326 Bydgoszcz, POLAND, phone: +48 523749014, fax: +48 523749005, e-mail: lukas@utp.edu.pl

A) B)

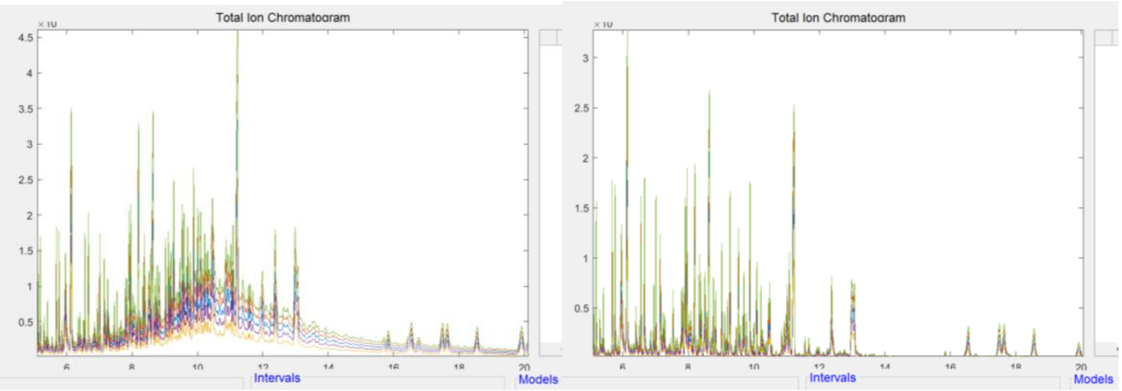

Figure S1. Effect of denoising filter on chromatograms: five chromatograms (sample S3) imported as CDF files into PARADISe program A) without the denoising filter B) with the denoising filter applied earlier.
